# Gene by environment QTL mapping through multiple trait analyses in blood pressure salt-sensitivity: identification of a novel QTL in rat chromosome 5

**DOI:** 10.1186/1471-2350-7-47

**Published:** 2006-05-22

**Authors:** Júlia M Pavan Soler, Alexandre C Pereira, César H Tôrres, José E Krieger

**Affiliations:** 1 Mathematics and Statistics Institute, University of São Paulo, Brazil; 2 Laboratory of Genetics and Molecular Cardiology, Heart Institute, University of São Paulo, Brazil

## Abstract

**Background:**

The genetic mechanisms underlying interindividual blood pressure variation reflect the complex interplay of both genetic and environmental variables. The current standard statistical methods for detecting genes involved in the regulation mechanisms of complex traits are based on univariate analysis. Few studies have focused on the search for and understanding of quantitative trait loci responsible for gene × environmental interactions or multiple trait analysis. Composite interval mapping has been extended to multiple traits and may be an interesting approach to such a problem.

**Methods:**

We used multiple-trait analysis for quantitative trait locus mapping of loci having different effects on systolic blood pressure with NaCl exposure. Animals studied were 188 rats, the progenies of an F2 rat intercross between the hypertensive and normotensive strain, genotyped in 179 polymorphic markers across the rat genome. To accommodate the correlational structure from measurements taken in the same animals, we applied univariate and multivariate strategies for analyzing the data.

**Results:**

We detected a new quantitative train locus on a region close to marker R589 in chromosome 5 of the rat genome, not previously identified through serial analysis of individual traits. In addition, we were able to justify analytically the parametric restrictions in terms of regression coefficients responsible for the gain in precision with the adopted analytical approach.

**Conclusion:**

Future work should focus on fine mapping and the identification of the causative variant responsible for this quantitative trait locus signal. The multivariable strategy might be valuable in the study of genetic determinants of interindividual variation of antihypertensive drug effectiveness.

## Background

The genetic mechanisms underlying interindividual blood pressure variation reflect the complex interplay of both genetic and environmental variables. Because of the considerable health and economic costs of hypertension, the identification of the genetic determinants of blood pressure homeostasis represents a fundamental step towards more cost-effective and specific approaches to this public health problem.

Studies of the genetic basis of hypertension have identified multiple quantitative trait loci (QTL), especially in rat models [1]. Previously, we described 5 different chromosomal loci that, collectively, explain 43% of the total systolic blood pressure variation exhibited among an F2 progeny from a cross between the Brown-Norway and spontaneously hypertensive rat strains [2]. However, despite the intense efforts being undertaken to identify QTL participating in blood pressure regulation, few studies to date have focused on the search for and understanding of QTL responsible for gene × environmental interactions.

In this respect, statistical methods for the detection of genes influencing QTL with the aid of genetic markers are well developed for the analysis of a single trait and have become the current standard methods. However, they may not be the most sensitive statistical approach when gene × environmental models are studied. Composite interval mapping [3] combines maximum likelihood interval mapping with multiple regression using marker cofactors, which increases the power of QTL detection and allows the simultaneous study of more than one QTL in the genome. The principle of composite interval mapping has recently been extended to multiple traits [4], enabling the evaluation of the main QTL effects, as well as QTL by trait and QTL by environmental interactions [5].

Genotype by environmental interaction is the differential expression, or effect, of a gene in different environments. Multitrait analysis has a number of intrinsic properties that are particularly interesting when one is concerned with the biological nature of these interactions [6]. One of the reasons is because, in practice, experimental designs are based on the paired comparison of the same set of genotypes recorded on markers evaluated phenotypically in 2 or more different environments, for example before and after salt loading. Therefore, because of the extensive covariance between these traits, multiple trait analysis is predicted to be more sensitive than single-trait QTL analysis is in identifying QTL important for treatment of response or sensitivity to environmental insults, or both.

In this study, we used multiple trait analyses for QTL mapping of loci involved in both blood pressure salt-sensitivity response in the progenies of an F2 rat intercross between the hypertensive strain, spontaneously hypertensive rat (SHR), and the normotensive strain, Brown-Norway (BN).

## Methods

### Animals

We used the same phenotypic databank described by Schork [2]. Briefly, the study examined blood pressure variation before and after a salt-loading experiment in intercross (F2) progeny from a cross between the BN normotensive rat strain and the SHR; 235 rats were available for study. They were phenotyped 16 weeks after birth over a 14-day period. Baseline levels of systolic blood pressure (SBP), diastolic blood pressure (DBP), mean arterial blood pressure (MAP), and heart rate (HR) were measured directly through an indwelling catheter in the femoral artery. Immediately after baseline parameters were established, the rats were given a diet that included water with 1.0% NaCl for 13 days. At the end of 13 days, each rat was anesthetized and a catheter was placed in its contralateral femoral artery. SBP, DBP, MAP, and HR were remeasured on the day after this catheter was inserted (these new phenotypes were named SBPS, DBPS, MAPS, and HRS). Of the 222 rats completing the protocol, 188 were then genotyped for 179 markers following a standard protocol [2].

### Genetic marker map

After completion of the first genotyping effort, 179 markers were available for analysis. Although no new genotypes were available for this study, placement of these markers in the rat genome was considerably more precise. We reaccessed the genetic distances of each genotyped marker through sequential consultation of the following Internet databases: Rat Genome Database [7]; RatMap [8]; and Whitehead Institute – Rat Genome [9].

### Statistical methods

For crosses between inbred lines with multiple traits, mapping of QTL can be performed for each trait one at a time, based on the composite interval mapping method [10,3], or jointly on both traits, using the extended version in terms of multivariate regression models [4].

Likelihood approaches have been proposed for multiple trait analysis. Based on an extension of the composite interval mapping method, Jiang and Zeng [4] obtained the likelihood function in terms of a mixture of multivariate normal distributions. They considered an ECM algorithm to obtain the maximum likelihood estimates. The results were obtained by using QTL Cartographer software, [11]. In another context, Knot and Haley [12] described a multitrait least-squares analysis allowing a multivariate normal distribution to model the matrix containing the trait values for all individuals. In this study, we used the statistical approach of Jiang and Zeng [4].

In spite of several strategies that can be proposed to determine the genetic architecture of multiple correlated traits, it is not clear whether one should start with one multiple trait analysis or with a series of single trait analyses. For the analytic strategy adopted to determine QTL involved in NaCl responsiveness, the difference between the values of SBPS and SBP of each animal (DIF) denotes the phenotype. We performed 4 methods of QTL mapping: separate mapping for each SBP, SBPS, and DIF, and joint mapping for SBP and SBPS.

Let *y*_*SBP *_= (*y*_1*SBP*_, *y*_2*SBP*_,..., *y*_*nSBP*_)^*t *^and *y*_*SBPS *_= (*y*_1*SBPS*_, *y*_2*SBPS*_,..., *y*_*nSBPS*_)^*t *^be the trait vectors for systolic blood pressure before and after salt loading, measured in *n *F2 animals, respectively. In addition, let

*y*_*DIF *_= (*y*_1*SBPS *_- *y*_1*SBP*_, *y*_2*SBPS *_- *y*_2*SBP*_, ..., *y*_*nSBPS *_- *y*_*nSBP*_)^*t *^be the corresponding trait vector obtained through the difference between the SBPS and SBP measurements. For univariate composite interval mapping, the following model was considered the *k*-th trait evaluated in the *j*-th animal:



where for *k*-th trait *b*_*ok *_is the mean effect;  and  are the additive and dominant effects of the putative QTL;  and  are calculated through the markers of genotype information flanking putative QTL and the associated recombination frequencies; *x*_*kl *_and *z*_*kl *_are corresponding variables for marker *l *assuming *t *markers are used as genetic background controls; *b*_*kl *_and *d*_*kl *_are regression coefficients of the trait *y*_*kj *_on *x*_*kl *_and *z*_*kl*_, respectively; and *e*_*kj *_is normally distributed with mean 0 and variance . For simplicity, here we assume the same background genetics for all traits considered in the univariate analysis (SBP, SBPS, and DIF). Note that the model formulated in terms of variable DIF is proposed to accommodate the pre- and postsalt measurement dependence structure. By taking a putative QTL into account, the corresponding additive and dominant effects for the *k-th *trait can be searched for by testing the hypotheses

*H*_*o *_:  =  = 0; *H*_1 _: *at least one of them is not zero*.

Alternatively, the additive (dominant) effect can be tested under a non-null dominant (additive) effect. The test is performed with a likelihood ratio statistic (LR) or in terms of lod score statistics (0.217 LR).

If phenotypes *y*_*SBP *_and *y*_*SBPS *_are correlated, univariate analysis of the individual traits disregards the additional information implicit in the correlational structure. A bivariate analysis in which this phenotypic correlation is explicitly modeled will exploit more of the information content of the data, will increase power for detection of QTL and will improve precision of parameter estimates. In this context, the covariance between the error terms, *Cov *(*e*_*jSBP*_, *e*_*jSBPS*_) = *σ*_12 _= *ρ*_12_*σ*_*SBP *_*σ*_*SBPS*_, can be introduced in the analysis through the following model for the vector *y*_*j *_= (*y*_*jSBP*_, *y*_*jSBPS*_)^*t *^:



where, for likelihood analysis, the residual vector *e*_*j *_is assumed to be bivariate normally distributed with mean zero and variance-covariance matrix given by



For the n vectors *y*_*j *_= (*y*_*jSBP*_, *y*_*jNaSBP*_)^*t*^, Jiang and Zeng [4] define the likelihood function in terms of a mixture of bivariate normal distribution as



where *p*_2*j*_, *p*_1*j *_and *p*_0*j *_denote the prior probability of  taking values 2, 1, and 0, respectively, for the 3 possible genotypes of the putative QTL, and *f*_2_(*y*_*j*_), *f*_1_(*y*_*j*_) and *f*_0_(*y*_*j*_) represent the corresponding bivariate normal density function of the random vector *y*_*j*_. Maximum likelihood estimates of the parameters are computed through ECM algorithms, a special version of general EM algorithms [4].

Given a putative QTL, under joint mapping, the hypotheses to be tested are

*H*_1 _: *at least one of them is not zero*.

When joint mapping for QTL of 2 traits is indicated, statistical tests can proceed to test whether the QTL has effects on either one or both of the 2 traits:



and



The estimates of model parameters with and without constraints can be obtained by numerical procedures using the ECM algorithm, and the likelihood ratio test statistics can then be calculated.

Jiang and Zeng [3] and Mangin et al [13] discuss the relative advantage of the joint analysis as compared with separate analyses. When the correlation is null (ρ = 0), the test statistics under the multivariate model are approximately the sum of those under the corresponding univariate models. Otherwise, joint test statistics can be smaller or larger than the sum of separate test statistics, depending on the sign and magnitude of the residual correlation and the differences between QTL effects. The power of the joint analysis can increase significantly if the product of QTL effects and the residual correlation are in different directions.

## Results and discussion

The empirical correlation coefficients of the systolic and diastolic blood pressure measurements evaluated before and after salt intake are shown in Table 1.

**Table 1:** Empirical correlation coefficient to the F2 inbred study.

Condition	SBP-DBP	SBPS-DBPS	SBP-SBPS	DBP-DBPS
Correlation	0.902	0.908	0.423	0.445

**Figure 1:**
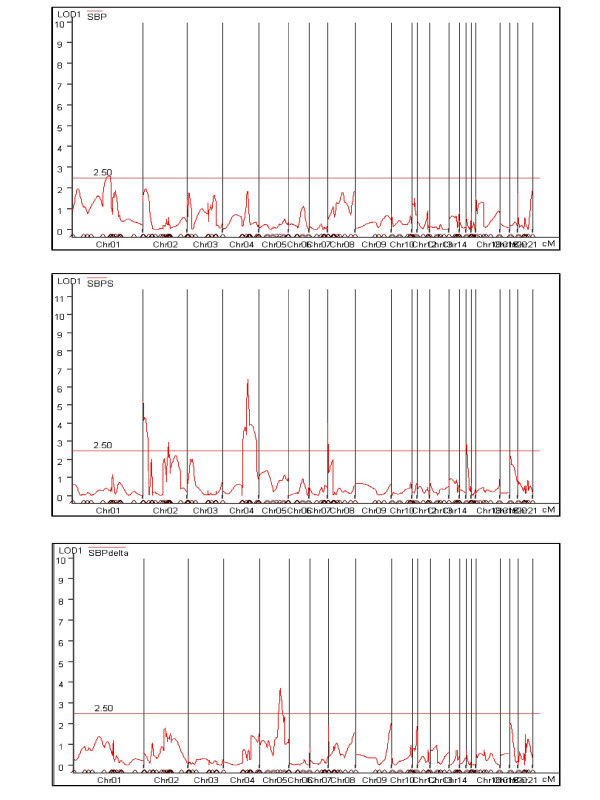
Lod Score statistics under univariate model adjustment for SBP, SBPS and DIF.

**Figure 2:**
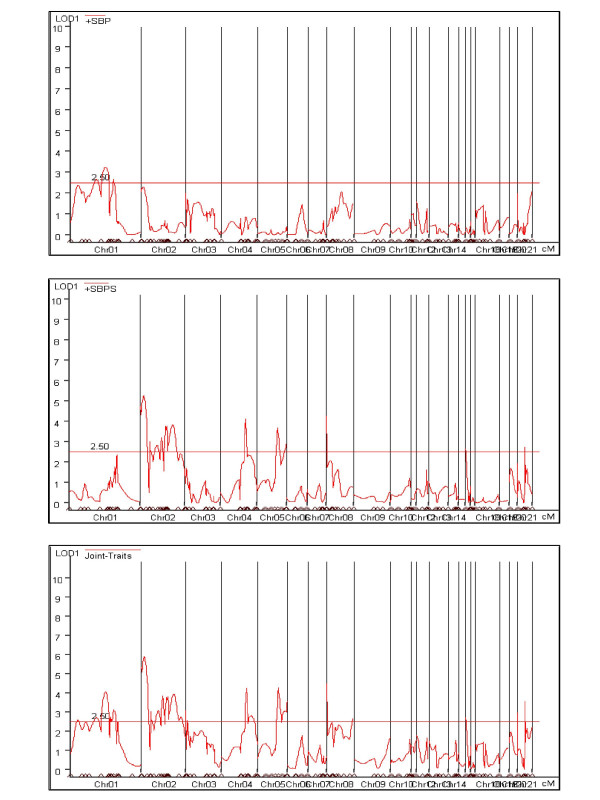
Lod Score statistics for the joint effects under the multivariate model.

Pleiotropic studies can be conducted exploring the correlation between systolic and diastolic measurements in each condition, before and after salt intake. The highest correlation values (*r *= 0.902 and *r *= 0.908) were obtained in these situations. In addition, gene-environment interaction studies explore the correlation between the blood pressure values taken before and after salt intake. Considering this last alternative, we adjusted the statistical models shown previously for traits SBP and SBPS. The linkage signals for putative QTL fixed on the marker map along the rat 21 chromosome are in Figures 1 and 2 for univariate and multivariate adjustments, respectively. In Tables 2 and 3, the corresponding estimated QTL effects and likelihood ratio test statistics are showed.

**Table 2:** Likelihood ratio statistics, additive and dominant effect estimates for the putative QTL under univariate models with higher linkage signal in the chromosome 5.

Variable	LR	Additive Effect	Dominant Effect
SBP	1.2483	-1.3033	2.5755
SBPS	3.8945	3.0523	-1.4667
DIF	17.1330	8.0698	-8.6078

**Table 3:** Likelihood ratio statistics, additive and dominant effect estimates for the putative QTL under multivariate model with higher linkage signal in the chromosome 5.

Variable	LR	Additive Effect	Dominant Effect
SBP	1.2147	-0.64828	2.10244
SBPS	16.8486	4.21706	-5.99663
Joint	19.6925		

Separate mapping for SBP, SBPS in Figure 1 do not identify any new linkage signal in the rat genome other than the ones previously published for the SBPS trait [2]. Previous regions already identified in chromosomes 2, 4, and 8 for the SBPS trait have been thoroughly discussed by Schork et al [2]. Interestingly, a univariate approach using the DIF phenotype identified a new linkage signal in one position on chromosome 5 (close to marker R589). For univariate analysis, this QTL was only revealed when trait DIF was analyzed. On the other hand, this significant signal was confirmed through multivariate mapping by using both the SBP and the SBPS traits (Figure 2).

QTL effect estimates for this position shown in Tables 2 and 3 indicate different directions for additive and dominant terms, changing the corresponding signals for each SBP and SBPS trait. In this context, because the residual correlation between the traits is positive (*r *= 0.423), analytically, we are under the most favorable situation to increase the power of the joint analysis as compared with separate analyses. The model obtained of variable DIF in terms of the models formulated for SBP and SBPS can be clarified. The corresponding regression coefficients associated with additive and dominant effects for variable DIF are illustrated by



Because  and  are in different directions, a significant linkage signal may be identified only for the adjustment of the variable DIF.

To understand the genetic architecture controlling the responsiveness to salt intake due to any gene on chromosome 5, close to marker R589, note that the results presented in Tables 2 and 3 suggest a modest (but nonsignificant) positive dominant effect on SBP and a more expressive (and significant) negative dominant effect on systolic blood pressure levels with salt intake (SBPS levels).

One potential study limitation is the number of genetic markers used in the genome-mapping experiment. Although a large number of F2 analyses have been reported [1], genes responsible for SHR hypertension have not yet been identified. One of the possible reasons for this is the small number of markers genotyped in these studies. In our mapping experiment, we used 179 markers, polymorphic between the SHR and BN strains. Although genetic distances and locations of these markers were updated before conducting our analysis, it is possible that QTL important for this gene-environment interaction are still unidentified.

## Conclusion

We used univariate and multivariate mapping models for detection of genes putatively relevant to the systolic blood pressure variation due to salt loading. With this approach, a new and relevant QTL involved in the responsiveness to salt was identified on a region close to marker R589 in chromosome 5 of the rat genome. With a univariate approach, the reduction of the 2-trait analysis to the difference between SBP measurements was sufficient to clarify the interchanges on the genetic effect direction before and after salt intake. The use of a multivariate approach was more sensitive and disclosed the new chromosome 5 QTL by using both the SBP and SBPS traits without the need of any summary measurement. Future work should focus on fine mapping and the identification of the causative variant responsible for this QTL signal.

The multivariable strategy might be valuable in the study genetic determinants of interindividual variation of antihypertensive drug effectiveness.

## Competing interests

The author(s) declare that they have no competing interests.

## Authors' contributions

JMPS and ACP performed the statistical analysis, conceived the study, and drafted the manuscript. CHT participated in genotype data generation and genetic map construction. JEK helped conceive the study and participated in its design and coordination. All authors have read and approved the final manuscript.

## Pre-publication history

The pre-publication history for this paper can be accessed here:


